# Supplementary material for: Symbionts with eroded genomes adjust gene expression according to host life-stage and environment
Source: EMBO Rep. 2025 Aug 8;26(19):4656–74. doi: 10.1038/s44319-025-00525-2 (PMC12508126; doi:10.1038/s44319-025-00525-2)
Supplement: Supplementary file 17 — Expanded View Figures [file 44319_2025_525_MOESM17_ESM.pdf]

Expanded View Figures

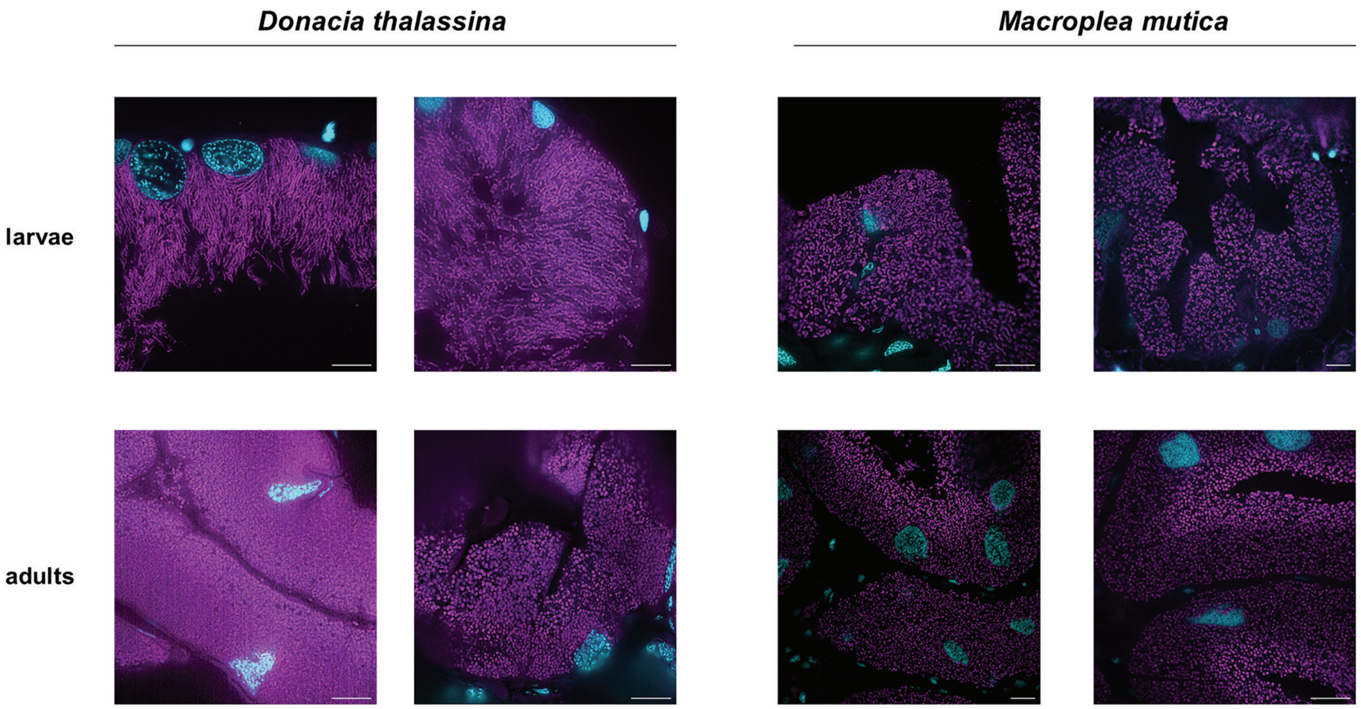

Figure EV1. FISH for symbDTHA and symbMMUT in larvae and adults of the respective hosts.

Symbionts in the symbiotic organs of 2 larvae and 2 adults of *D. thalassina*, and 2 larvae and 2 adults of *M. mutica*. Overlay of a probe specific to Donaciinae symbionts (magenta) and DNA staining with DAPI (cyan). Scale bars correspond to 20 µm. Raw datasets can be found in https://doi.org/10.17617/3.VQABI2.

© The Author(s)
